# Fancy a gene? A surprisingly complex evolutionary history of peroxiredoxins.

**DOI:** 10.15698/mic2015.02.189

**Published:** 2015-01-28

**Authors:** Alena Zíková, Miroslav Oborník, Julius Lukeš

**Affiliations:** 1 Institute of Parasitology, Biology Centre, Czech Academy of Sciences, 370 05 České Budějovice (Budweis), Czech Republic.; 2 Faculty of Science, University of South Bohemia, 370 05 České Budějovice (Budweis), Czech Republic.; 3 Institute of Microbiology, Czech Academy of Sciences, 379 81 Třeboň, Czech Republic.; 4 Canadian Institute for Advanced Research, Toronto ON, M5G 1Z8, Canada.

**Keywords:** horizontal gene transfer, Apicomplexa, endosymbiont, Plasmodium, Chromera, peroxiredoxin, oxidative stress

## Abstract

While the phylum Apicomplexa includes “only” several thousand described species of obligatory parasites of animals, it may in fact be the most specious group of parasitic protists with over a million species 1. The best known representatives are *Plasmodium *spp., *Toxoplasma gondii* and *Cryptosporidium *spp., which belong to the most important and widespread human parasites exacting an enormous disease burden. On the other hand, dinoflagellates and colpodellids, which are monophyletic with the apicomplexans, are ecologically highly significant, as they belong to the most abundant marine protists 2. As the common ancestor of these groups was most likely a free-living photosynthesizing protist, one wonders, which evolutionary forces contributed to the dramatic transition of some of its descendants into the arguably most successful intracellular parasites? Although a range of various processes and mechanisms contributed to this transition, most likely it also involved an acquisition of genes via horizontal gene transfer (HGT), which might have provided typical characteristics of a parasitic cell, such as immune escape, nutritional dependence and the capacity to invade other cells.

HGT is a movement of DNA between two different organisms that allows them to gain novel features different from those which can be passed from a parent to an offspring. Being most widespread in eubacteria, it impacts the composition of their genomes to such an extent that multigene phylogenetic analyses form a net instead of a tree, demonstrating their extreme mosaicism. HGT also played the most crucial role in eukaryotic evolution, as it enabled gene transfer from endosymbionts to the host nucleus, contributing to the emergence of extant mitochondria and plastids. As a result of this development, organellar genomes encode only a small fraction of organellar proteins, with the vast majority of them being encoded by the nuclear genome. The proteins are then post-translationally imported to the organelles using complex targeting signals 3.

Moreover, random HGTs from a prokaryote to a eukaryote have been claimed for unicellular as well as for multicellular eukaryotes 3. Indeed, the number of HGT events documented from the latter hosts is steadily growing and some recently described cases, such as the independent HGTs of antibacterial toxins to supplement immune system of higher eukaryotes, are truly stunning 4,5. Still, it is protists, such as the diatom *Phaeodactylum tricornutum* 6 and rumen-dwelling ciliates, that truly excel in their capacity to acquire genes of prokaryotic provenance 7. When focusing on parasitic protists, the cases of prokaryote-eukaryote HGTs, resulting in the increase of fitness, obviously stand out. The prominent ones include the transfer of γ-proteobacterial N-acetylneuraminate lyase, which boosts nutrient uptake from the vertebrate host into the parabasalid *Trichomonas vaginalis*, and the acquisition of γ-proteobacterial thymidine kinase by the apicomplexan *Cryptosporidium parvum* that is responsible for salvaging pyrimidine nucleotides from the host. Another example is the capacity of the microsporidium *Antonospora locustae* to employ a bacterial class II photolyase to repair DNA damage caused by ultraviolet radiation 8. Rarely, several components of a single metabolic pathway have been acquired either separately or at once, but from a single source, as seems to be the case of the kinetoplastids *Leishmania* spp., originally deficient of heme synthesis, which obtained enzymes for the last three steps of the pathway from a γ -proteobacterium via HGT 9.

Another example of a prokaryote-eukaryote HGT is brought by a recent study by Djuika *et al.* that characterizes a special subclass of peroxiredoxin 5 (Prx5) isoform in *Plasmodium falciparum *10. This parasite has a complex life cycle involving a mosquito vector and a vertebrate host. Causing malaria, *P. falciparum* and related species spend part of their life cycle inside of human or other vertebrate erythrocytes, where they are exposed to high levels of oxidative stress from host defense reactive oxygen and reactive nitrogen species. Even though *P. falciparum* possesses a superoxid dismutase, it lacks catalase and glutathione peroxidase and thus its redox system relies heavily on cysteine-dependent peroxidases. *P. falciparum* contains 5 peroxidases found in different cell compartments: namely, Prx1 and Prx6 were localized to the cytosol, Prx1m to the mitochondrion, PrxQ to the nucleus and Prx5 (also called antioxidant protein, PfAOP) to the apicoplast 11. The apicoplast is a non-photosynthetic plastid, acquired by secondary endosymbiosis of a rhodophyte, which retains several essential processes, such as biosynthesis of fatty acids, heme, isoprenoids and iron-sulfur clusters 12. A PfAOP localization to the apicoplast was experimentally proven by fusing its N’ terminus with green fluorescent protein 13. However, by careful examination of cells stained with specific anti-PfAOP antibodies, Djuika *et al.* reports a dual localization of this protein, supplemented by immunoblot analysis of subcellular fractions of the *P. falciparum* cells. This interesting observation was further corroborated by experiments with three different PfAOP-GFP chimeras 10.

With more careful examinations of intracellular localization using various combinations of protein tags, antibodies and fractionations, dual localization of proteins is emerging as a surprisingly frequent phenomenon than appreciated until recently. To increase usefulness of a given protein, at least in some cases, it is advantageous for the eukaryotic cell to target it to more than one compartment. Particularly in *P. falciparum*, three different proteins (lipoate protein ligase A2 and isoforms of serine hydroxymethyltransferase) were shown to be dually targeted to the apicoplast and the mitochondrion 14,15. Moreover, glutathione peroxidase-like thioredoxin peroxidase seems to be targeted to even three compartments, namely both organelles and the cytosol 16. The cytosolic/apicoplast co-localization was also evidenced for other two anti-oxidant redox enzymes, glutathione reductase and thioredoxin reductase 13.

Multiple targeting can be attained by either alternative translation initiation or alternative transcription. Interestingly, Djuika *et al.* reports another mean of dual localization that is reflected by modular gene architecture, in the case of PfAOP constituted by two exons. While exon 1 encodes the bipartite leader sequence consisting of ER-type signal peptide followed by a transit peptide required for import into the apicoplast, exon 2 specifies the actual enzymatically active Prx5 domain. Remarkably, such a modular architecture is specific only for *Plasmodium* species. As the acquisition of a transit peptide by exon shuffling seems to be an easy way how to acquire the apicoplast localization 17, the question remains why was this solution explored exclusively by the malaria parasites, and what are the physiological advantages of PfAOP being dually localized.

Another significant finding of Djuika *et al.* lies in the phylogenetic origin of apicomplexan Prx5. Since PfAOP contains the bipartite targeting sequence, its origin by secondary endosymbiosis is appealing. However, the authors nicely showed that this gene was acquired by a prokaryote-eukaryote HGT. Moreover, they offer an attractive hypothesis of gene transfer between a marine bacterium and an apicomplexan ancestor. In such case, one would expect that the phylogenetic analysis of Prx5 homologs from free-living chromerid algae will shed light on this interesting issue.

Therefore, we have searched the draft genome sequences (our unpublished data) of two photosynthetic predecessors of *Plasmodium* and other apicomplexans, *Chromera velia* 18 and *Vitrella brassicaformis *19, and for each of them we found 5 genes encoding proteins homologous to *Plasmodium* Prx5. The correspondingly complemented dataset was analyzed by Bayesian inference (Fig. 1) and maximum likelihood (tree not shown). Indeed, two genes from *Chromera* (Cvel_16952.t1 and Cvel_15038.t1) and a single one from *Vitrella* (Vbra_19296.t1) appear to be of bacterial origin. However, while Cvel_16952.t1 branches between cyanobacteria and γ-proteobacteria, Cvel_15038.t1 and Vbra19296.t1 together constitute a yet different branch within the bacterial clade (Fig 1). It should be noted that the tree is relatively poorly supported and hence does not allow exact specification of particular bacterial gene donors. Prediction programs revealed the presence of a mitochondrial signal peptide in the bacterial protein of *Vitrella* and in a distantly related homolog in *Chromera*, which is also clearly of bacterial origin (Fig. 1). However, in addition to bacterial Prx5, also homologs of undisputable eukaryotic origins were found in both chromerids. The Cvel_31961.t1 gene, supposed to be of exosymbiont origin and confined to *Chromera*, constitutes a clade together with fungi and the early-branching apicomplexan *Gregarina niphandroides*. The supposedly endosymbiont eukaryotic counterparts of *Chromera* (Cvel_19916.t1 and 16350.t1) and *Vitrella* (Vbra_5437.t1, Vbra_7622.t1, Vbra_2354.t1 and Vbra_19764.t1) form a compact clade in the proximity of green algae and plants. While this gene was obviously duplicated in both chromerids, in *Vitrella* it went through the duplication event twice (Fig. 1). To our surprise, the supposedly exosymbiont-derived protein (Cvel_31961.t1) carries a bipartite targeting sequence, predicted with high confidence at its N-terminus, and is thus likely plastid-targeted. The second eukaryotic Prx5 from *Chromera* (Cvel_19916.t1) possesses a mitochondrial signal peptide. Two of the eukaryotic genes from the endosymbiont of *Vitrella* (Vbra_5437.t1 and Vbra_7622.t1) encode bipartite targeting sequences with corresponding proteins likely to be delivered to the plastid. Finally, yet another eukaryotic Prx5 is predicted to be targeted to the mitochondrion of *Vitrella* (Fig. 1).

**Figure 1:**
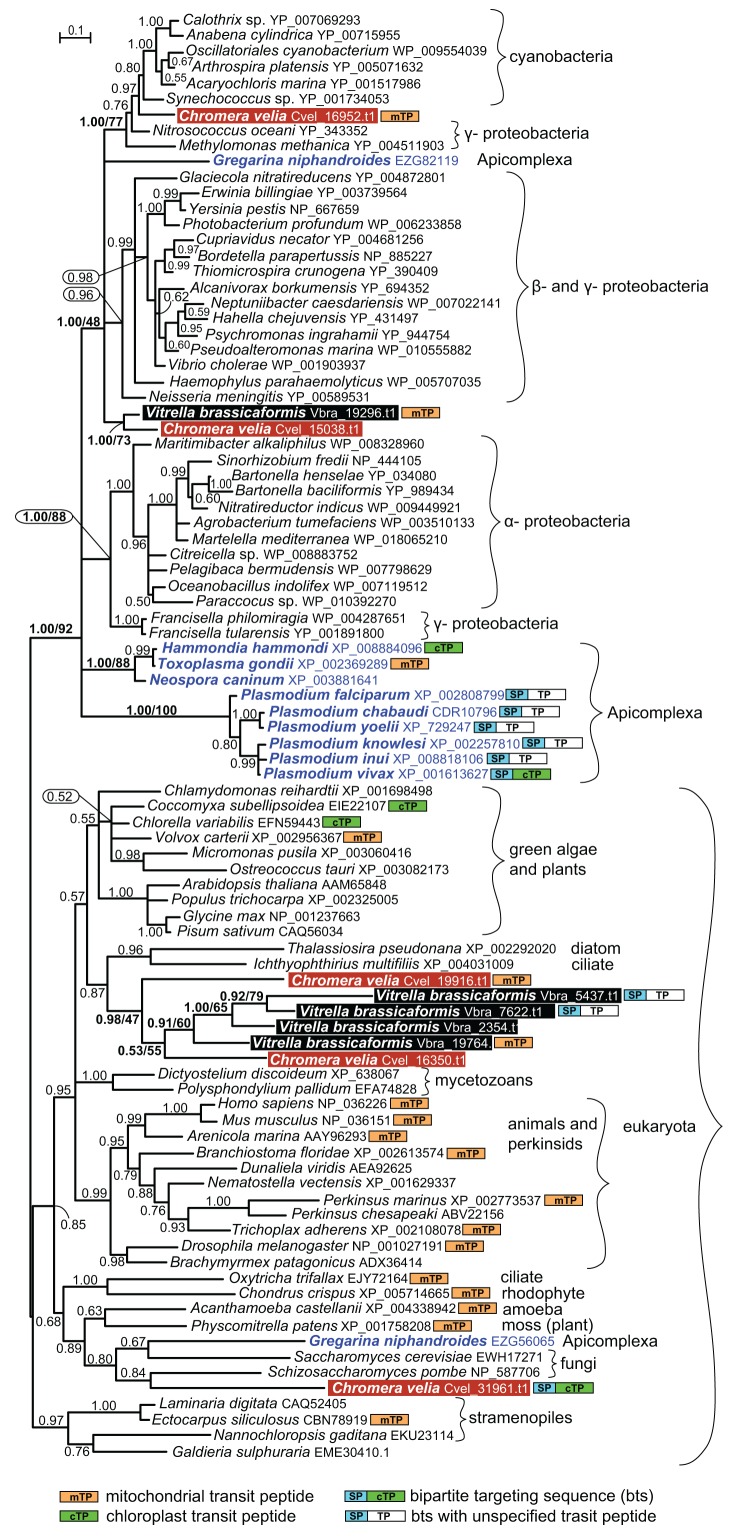
FIGURE 1: Bayesian phylogenetic tree as inferred from Prx5 amino acid sequences. The original dataset used by Djuika *et al.* was extended by the addition of relevant sequences from the chromerid algae *Chromera velia* and *Vitrella brassicaformis*, and the early-branching apicomplexan *Gregarina niphandroides*. Sequences were aligned using MAFFT, edited by Gblocs (both implemented in SeaView), and the alignment was analyzed by Bayesian inference and maximum likelihood (ML) method. MrBayes 3.2 20 was used to assess Bayesian topologies and posterior probabilities (PP) using WAG model. Two independent Monte-Carlo Markov chains were run under the default settings for 3 million generations; we used 500,000 generations as a burn-in and omitted them from topology reconstruction and PP calculation. ML bootstrap support is shown for selected nodes in the tree; ML tree was computed by RAxML 21, using γ corrected WAG model with bootstrap analysis being inferred from 1,000 replicates. Putative intracellular localizations of the Prx5 proteins were predicted using the TargetP and SignalP programs, respectively 22.

Based on our tree we suggest that the phototrophic apicomplexan ancestor evolved its own broad repertoire of the Prx5 proteins. While the *Chromera* plastid seems to import the original exosymbiont protein, the mitochondrion likely uses at least two Prx5 proteins, namely the eukaryotic one acquired during secondary endosymbiosis, and the prokaryotic version obtained from a bacterium. In addition to these genes, the ancestral apicomplexan also acquired more Prx5 genes from various bacteria, most likely by several independent HGT events. During the transition of a phototroph into a parasite, all Prx5 proteins of eukaryotic origin were lost, with a single bacterial gene being retained in the apicomplexan genome.

The Prx5 gene found in *P. falciparum* is, however, very probably unrelated to the bacterial homologues found in both chromerid algae. At least two independent acquisitions of a prokaryotic Prx5 via HGT in *Chromera* make an additional event in Apicomplexa quite likely. Moreover, the early-branching apicomplexan *G. niphandroides* contains yet another bacterial Prx5, which seems to be unrelated to its homologs in *Toxoplasma*, *Neospora* and *Plasmodium* (Fig. 1). The emerging scenario is thus a complex one. At this point, we cannot distinguish between multiple independent acquisitions of Prx5 in various apicomplexan lineages and the presence in the apicomplexan ancestor of several prokaryotic Prx5 genes, which were gradually lost in the derived lineages.
